# Patterns of dairy food intake, body composition and markers of metabolic health in Ireland: results from the National Adult Nutrition Survey

**DOI:** 10.1038/nutd.2016.54

**Published:** 2017-02-20

**Authors:** E L Feeney, A O'Sullivan, A P Nugent, B McNulty, J Walton, A Flynn, E R Gibney

**Affiliations:** 1 UCD Institute of Food and Health, Science Centre South, University College Dublin, Dublin, Ireland; 2 Food for Health Ireland, University College Dublin, Dublin, Ireland; 3 School of Food & Nutritional Sciences, University College Cork, Cork, Ireland

## Abstract

**Background:**

Studies examining the association between dairy consumption and metabolic health have shown mixed results. This may be due, in part, to the use of different definitions of dairy, and to single types of dairy foods examined in isolation.

**Objective:**

The objective of the study was to examine associations between dairy food intake and metabolic health, identify patterns of dairy food consumption and determine whether dairy dietary patterns are associated with outcomes of metabolic health, in a cross-sectional survey.

**Design:**

A 4-day food diary was used to assess food and beverage consumption, including dairy (defined as milk, cheese, yogurt, cream and butter) in free-living, healthy Irish adults aged 18–90 years (*n*=1500). Fasting blood samples (*n*=897) were collected, and anthropometric measurements taken. Differences in metabolic health markers across patterns and tertiles of dairy consumption were tested via analysis of covariance. Patterns of dairy food consumption, of different fat contents, were identified using cluster analysis.

**Results:**

Higher (total) dairy was associated with lower body mass index, %body fat, waist circumference and waist-to-hip ratio (*P*<0.001), and lower systolic (*P*=0.02) and diastolic (*P*<0.001) blood pressure. Similar trends were observed when milk and yogurt intakes were considered separately. Higher cheese consumption was associated with higher C-peptide (*P*<0.001). Dietary pattern analysis identified three patterns (clusters) of dairy consumption; 'Whole milk', 'Reduced fat milks and yogurt' and 'Butter and cream'. The 'Reduced fat milks and yogurt' cluster had the highest scores on a Healthy Eating Index, and lower-fat and saturated fat intakes, but greater triglyceride levels (*P*=0.028) and total cholesterol (*P*=0.015). conclusion: Overall, these results suggest that while milk and yogurt consumption is associated with a favourable body phenotype, the blood lipid profiles are less favourable when eaten as part of a low-fat high-carbohydrate dietary pattern. More research is needed to better understand this association.

**Conclusion:**

Overall, these results suggest that although milk and yogurt consumption is associated with a favourable body phenotype, the blood lipid profiles are less favourable when eaten as part of a low-fat high-carbohydrate dietary pattern. More research is needed to better understand this association.

## Introduction

The topic of dairy food consumption and its relationship to metabolic health is controversial.^1^ Dairy products, particularly higher-fat dairy products such as cheese, butter, cream and full-cream milk are considered significant sources of energy, and of saturated fat (SFA), contributing ~20% of dietary SFA intakes in Ireland^2^ and the in UK.^3^ Figures from the United States are similar; cheese alone contributes to 16.5% of SFA intakes, whereas milk contributes 8.5%, and margarine and butter (grouped together) contribute 5.8%.^4^ While many dairy products are considered energy dense, bovine milk is also well-recognised as an important contributor to nutrients in the human diet,^2, 5^ containing amino acids, fats and oligosaccharides, as well as a range of nutrients including calcium, magnesium, iodine, riboflavin, folate, B vitamins, vitamin A and vitamin E.^5^ Some dairy products, cheeses in particular, are known sources of bioactive peptides with varying functions.^6^ In addition, many dairy foods contain various other bioactive compounds, such as oligosaccharides, and sphingolipids.^7, 8^

Over 60% of the fat in dairy fat is saturated,^9^ yet reports suggest potential health benefits of dairy food consumption on a variety of aspects of metabolic health.^7, 9, 10, 11, 12, 13, 14^ These include: an association with reduced body mass index (BMI) and waist circumference;^10^ improvement of blood lipid profiles;^11, 12, 13^ reduced risk of hypertension;^14^ improvement of glycaemic responses^15^ and reduced risk of type 2 diabetes.^16, 17^ However, conflictions exist in the current literature, with a number of reports observing no association with dairy intake and various markers of metabolic health.^18, 19, 20, 21, 22, 23^ These different findings may be partly due to the variety of available dairy foods, and differing levels of fat within these.^24^ One particular issue is a lack of a standard definition of ‘high' and ‘low' fat dairy foods; with many studies grouping full fat or whole milk, cheeses, cream and butter into ‘high fat dairy'^24, 25^ while semi-skimmed and skimmed milk, and reduced fat yogurt are often grouped in ‘low fat dairy'.^24^ However, these groupings are not consistent, and vary from study to study. For example, Larsson *et al.*^26^ used a 3% fat cut-off point for ‘high fat' dairy; other researchers have used figures of 3.5%,^27^ while others do not detail the fat levels used for the determination of ‘high' and ‘low' fat dairy in their analyses.^25, 28^ Some researchers have used multiple cut-off values within the one analysis;^29, 30^ depending on the dairy product. This lack of a universal definition results in dairy foods being grouped differentially into ‘high' and ‘low' fat, depending on the study, and may impact results by obscuring potential differences in the effect of the varying fat content on markers of metabolic health. Further, categorisations based on fat content alone ignore the rest of the food ‘matrix' in which that fat is consumed. The food matrix describes foods in the context of both their structure, and their nutrient content, with the goal of understanding how these interact together. Recent research suggests that the overall food matrix in which a nutrient is consumed is an important consideration, as evidenced by different dairy foods (mainly butter vs cheese) with the same SFA content having quite different effects on blood lipids in randomised controlled feeding studies.^11, 13, 31, 32, 33^ When examining the link to health, intakes are often examined at a total dairy intake level, or considered by intakes of a specific dairy component, such as milk^34^ or cheese,^35^ without addressing the impact of the combinations of foods consumed in different dietary patterns. The use of dietary pattern analysis allows for the capture of natural eating patterns^36^ and thus affords a novel opportunity to incorporate the different fat contents of dairy foods into an analysis, and examine how they are eaten in combination, and how these relate to metabolic health. The Irish diet is relatively homogenous with most people consuming dairy foods,^37^ therefore allowing patterns of dairy product intake to be examined. Dietary intake in Ireland is consistent with the UK,^3^ with relatively high dairy intakes as observed in other Northern European countries.^38^

This paper aims to address knowledge gaps in the association of dairy food consumption and its relationship to metabolic health markers (Anthropometric measures, serum lipids, blood pressure, HOMA and QUICKI scores, and inflammatory cytokines) using different definitions of dairy. Associations will be examined using standard tertile analysis, and then using dietary pattern analysis, to incorporate a measure of the different types of dairy foods eaten, and the patterns in which they are consumed.

## Materials and methods

### Mean daily intakes of dairy foods

Dietary intake data were available for *n*=1500 people as part of the National Adult Nutrition Survey (NANS) conducted in the Republic of Ireland from 2008 to 2010.^39^ Ethical approval for the study was received from the Human Research Ethics Committee at the University College Dublin, and by the University College Cork Clinical Research Ethics Committee of the Cork Teaching Hospitals. Written, informed consent was obtained from participants before commencing the study, and participants were randomly selected from a national database to represent the population in terms of the urban–rural divide, age, sex and social class, based on the 2006 census, with a 59.6% response rate.^40^ Each food and beverage consumed over a 4-day period was recorded and entered into a semi-weighed food diary. In brief, participants were given a set of scales, trained in their use, and were requested to weigh all foods and beverages, whenever possible over a 4-day period. Leftovers were also recorded. For any items where weighing was not possible, an estimate was obtained via an interview, using a food portion size atlas, during one of the three visits made to participants by researchers over the 4-day collection period. Other details were also obtained including brand information, cooking methods and eating location. Full details of methodology are published elsewhere.^39^ Using the database of all foods consumed in the NANS, a mean daily intake (g per day) of total dairy foods consumed was calculated for each person, where intakes from discrete foods and composite dishes were included. Mean daily amounts (g per day) of each individual dairy food consumed were also calculated. All participants in the study consumed dairy in some form (either as discrete foods or as an ingredient) over the 4-day period. Tertiles of individual dairy food intakes were created. This was done by ranking participants based on their mean daily intake of each, and assigning them into ‘low', ‘medium' and ‘high' intake groups, and ‘non-consumers'. A healthy eating index (HEI) was created based on the alternate HEI (AHEI), as adapted by McCullough *et al.*^41^ In brief, the AHEI is a score based on nine components calculated from daily servings of fruit and vegetables, alcohol, nuts and soy, and cereal fibre, the ratio of red to white meat, the ratio of polyunsaturated fat to SFA, percentage energy from trans fat and duration of multivitamin use.^41^

### Identification of dairy patterns

To examine patterns of dairy intake by percentage energy contribution to diet, variables of mean daily intakes per MJ of energy intake were created for the main dairy foods consumed (milk, cheese, yogurt, butter and cream), and for each of the subtypes of dairy foods, based on fat content. These were: total milk per MJ, and subcategories whole milk, semi-skimmed milk, skimmed milk and fortified milk per MJ (all fortified milks consumed in this study were also reduced fat milks); total cheese per MJ and cheese subtypes (based on % fat in dry matter:^42^ skimmed milk cheeses, 0–10% fat; Partially skimmed milk cheeses, 10–20% fat; Medium-fat cheeses, 20–40% fat; Full-fat cheeses, 40–60% fat, and high-fat cheeses, 60% fat in dry matter or above). Total yogurt per MJ, total cream per MJ and total butter per MJ were also calculated.

On the basis of the frequency of consumption of each, some variables were collapsed into larger groups, and a final number of seven variables representing the different dairy foods consumed were used to identify patterns of dairy food consumption. These were: reduced fat milks, whole milk, higher-fat cheeses, lower-fat cheeses, total cream, total butter and total yogurt. The variables were transformed to standardised *z*-scores, and a two-step clustering approach^43^ was taken to identify patterns. Individuals were grouped into distinct, non-overlapping clusters, based on their dairy food consumption, using standardised *z*-scores for each dairy food type, to avoid differences simply due to variation in mean portion size. The procedure is considered suitable for large data sets^44^ and can be particularly useful in nutritional epidemiology to identify unique dietary exposure categories.^43^ In the first step, small pre-clusters are created based on a log-likelihood distance criterion. In the second step, these pre-clusters are merged into distinct dietary groups via agglomerative hierarchical clustering.^44^ A cross-validation was conducted on a random subsample (75%) of the group to validate the clusters.

### Biochemistry

A subset of the participants, *n*=1136 (75.7%) provided a blood sample. For the analyses presented here, only fasted serum samples were used. Samples were processed in laboratories in either University College Cork (UCC) or University College Dublin (UCD) within 5 h of collection by centrifuging at 1570 *g* for 15 min at 4 ^o^C. The resultant supernatant was stored at −80 ^o^C until time of analysis. A clinical bioanalyzer (RX Daytona, Randox Laboratories, Crumlin, County Antrim, UK) was used for triacylglycerol (lipase/glycerol kinase colorimetric); total cholesterol (cholesterol oxidase); high-density lipoprotein (HDL) (direct clearance) and glucose (glucose oxidase). A selection of pro- and anti-inflammatory cytokines was selected based on their association with metabolic health, and some of these were available as part of a Metabolic Array Kit (Randox Laboratories). Cytokines and hormones (TNF-α, interleukin2 (IL2), IL6, IL10, insulin, leptin and C-peptide) were measured via the biochip array system (Evidence Investigator, Randox laboratories). ELISA kits were used to measure leptin soluble receptor (R&D Systems, Oxon, UK) and adiponectin (ALPCO Diagnostics kit, Salem, NH, USA). All samples were run in duplicate and cytokine concentrations were calculated from a calibration curve. Standard quality control procedures were followed on both analysers to ensure data integrity. Intra- and interassay coefficients of variations were ⩽4.9% for triacylglycerol, ⩽4.1% for total cholesterol, ⩽6.2% for HDL-cholesterol, ⩽4.9% for glucose, ⩽7.1% for leptin soluble receptor, ⩽11.9% for adiponectin, ⩽7.7% for TNF-α, ⩽11.3% for IL2, ⩽10.9% for IL6, ⩽9.3% for IL10, ⩽18.5% for insulin, ⩽9.2% for leptin and ⩽11.7% for C-peptide.

### Anthropometry

Weight, percentage body fat, height, waist and hip circumference were measured by trained fieldworkers, according to standard operating procedures.^39^ Weight and percentage body fat was measured in duplicate using a Tanita SC-331S body composition analyzer (Tanita, Tokyo, Japan). Height was measured using the Leicester portable height measure to the nearest 0.1 cm. Waist and hip circumferences were measured in duplicate using a non-stretch tape to the nearest 0.1 cm. An average resting blood pressure was calculated from triplicate measurements, with 5-min intervals between each, using an Omron Series 5 blood pressure monitor (Omron Healthcare, Inc., 1200 Lakeside Drive Bannockburn, IL, USA).

### Statistical analyses

Differences in markers of metabolic health and differences in food and nutrient intakes between tertiles of dairy food intake and between clusters of dairy food intakes were analysed using analysis of covariance, adjusting for confounding factors, such as age, gender, BMI, HEI score and mean daily energy intake, where applicable. *X*^2^ analysis was used to examine differences in the ratio of males to females between clusters. Reporting bias was estimated using EI:BMR calculations and applying Goldberg's cut-off limits to identify misporting, calculated as 28·2% in this cohort, as previously reported.^40^ All analyses were conducted in SPSS v20 for Mac (IBM, New York, NY, USA).

## Results

A total of *n*=1500 people (740 m) aged between 18 and 90 years, took part of the study, across the age groups of 18–50, 51–64 and ⩾65 years (67.7%, 19.7% and 12.5%, respectively). About 70% of the sample reported living in an urban location, and 45% listed their occupations as professional, technical or managerial categories. The overall sample was representative of the Irish population,^39^ and fuller details on the demographics of the cohort have been published elsewhere, and are also available at www.iuna.net.

### Tertiles of total dairy consumption

The mean daily intake of total dairy (milk, cheese, yogurt, butter and cream) was 291.0 g per day (202.1 s.d). The group was divided into tertiles of total dairy intake, designated ‘High', ‘Medium' and ‘Low' (Table 1). ‘High' consumers of total dairy, after adjustment for energy intake, gender, age, social class and smoking, had significantly lower BMI and % body fat, (*P*<0.001), a lower waist circumference (*P*<0.001) and a higher insulin sensitivity score (*P*=0.001) compared with ‘low' consumers (Table 1). ‘High' consumers of dairy also had lower systolic (*P*=0.02) and lower diastolic blood pressure (*P*<0.001) and lower waist-to-hip ratio (*P*<0.001) compared with the ‘medium' and ‘low' consumer groups. There was no difference in fasting serum triglycerides, HDL-C or low-density lipoprotein-cholesterol across tertiles of total dairy intake. Of the blood biomarkers known to reflect aspects of metabolic health that were examined, there was no association between serum glucose, but serum insulin was significantly lower in the higher dairy intake groups (Table 1), and insulin sensitivity (as assessed via QUICKI) was significantly greater with increased dairy consumption (*P*=0.001). A number of other inflammatory markers were different across the tertiles; leptin was higher in low consumers of dairy, as was C-peptide (*P*=0.04). Adiponectin and leptin soluble receptor were both greater in the high dairy tertile (*P*=0.013 and 0.03, respectively) (Table 1).

### Tertiles of total milk, yogurt and cheese consumption

To further investigate dairy intakes, individuals were grouped into tertiles of total milk, total cheese and total yogurt intake separately (Supplementary Tables S1–S3). Increased total milk consumption was associated with a reduced BMI (*P*<0.001) and there was a trend towards higher muscle mass and lower body fat in the highest milk consumers *(P*=0.065 and *P*=0.076, respectively). Insulin was significantly greater in the lower milk tertile (*P*=0.02) and insulin sensitivity (QUICKI score) was greater in the higher consumers *(P*=0.002), whereas serum c-peptide significantly greater in the low consumers (*P*=0.02) (Supplementary Table S1). IL10 concentrations trended towards being lower in those with the greatest milk consumption (*P*=0.079).

When cheese consumption was examined separately, no differences were observed between the groups for any of markers of metabolic health except for C-peptide, which was greater with increasing cheese consumption (*P*=0.001) (Supplementary Table S2). High-yogurt consumers had significantly lower body fat (*P*=0.001), lower waist circumference (*P*=0.025) and lower waist-to-hip ratio (*P*=0.013) than any other tertiles. Serum concentrations of TNF-α were lower in the medium and high-yogurt consumers compared with the low and the non-consumers of yogurt also (*P*<0.001) (Supplementary Table S3).

### Patterns of dairy consumption

To example patterns of dairy consumption, cluster analysis was used. A two-step cluster approach resulted in a 3-cluster solution whereby each cluster exhibited a different dairy consumption profile. On the basis of the descriptive characteristics of the clusters (Table 2), the three clusters were named ‘Whole milk', ‘Butter and cream' and ‘Reduced fat milks and yogurt'. There was no significant difference in age across the clusters, but clusters were all significantly different in their mean daily total dairy intakes, energy and macronutrients. The proportion of males and females differed significantly across the clusters, *X*^2^(2)=43.2, *n*=1147, *P*<0.001. ‘Whole milk drinkers' had a greater proportion of men than women (M:F, 58:42), whereas there were more women in the ‘Reduced fat milks and yogurt' cluster (M:F, 41:59). The proportion of males to females in the ‘Butter and cream' cluster was more evenly spread at a ratio of 46:54 (Table 2).

To examine the effect of energy misreporting on the clusters, and as a further validation measure, the analysis was run again with the potential energy misreporters removed. Clustering on this smaller cohort (*n*=864) resulted in four clusters of dairy food intakes. These patterns were similar to those observed in the larger group, where all reporters were analysed, except that the ‘Reduced fat milks and yogurt' cluster appeared to form two separate, smaller clusters of individuals (of ‘Yogurt' and ‘Reduced fat milk'.

Cluster membership was broadly similar, with between 68 and 94% of people remaining in their original clusters; thus the remaining results are presented for the full cohort of people. (Of the 32% that moved from ‘Reduced fat milks and yogurt' they moved mainly into ‘Yogurt' or ‘Reduced fat milks', meaning that the patterns identified were still overall quite similar). Those in the ‘Whole milk' cluster consumed an average of 80.6 g of total fat daily, which was similar to the ‘Butter and cream' cluster (80.7 g per day, 26.8 g s.d.), but considerably higher than the 67.5 g fat per day consumed by the ‘Reduced fat milks and yogurt' cluster. The ‘Whole milk', and ‘Butter and cream' clusters also had similar intakes of SFA at 32.2 g per day each. Mean daily SFA intakes in the ‘Whole milk', and ‘Butter and cream' clusters were significantly greater than the ‘Reduced fat milks and yogurt' cluster, whose SFA intakes were 25.7 g daily, on average. Percent energy from SFA was also higher in these two groups than in the ‘Reduced fat milks and yogurt' cluster at 13.8% and 14% vs 12.2%.

Those in the ‘Reduced fat milks and yogurt' cluster were more likely to be female (*P*<0.001), and had a lower mean daily energy intake than the other two clusters (7.9 MJ vs 8.7 MJ and 8.8 MJ). The ‘Reduced fat milks and yogurt' cluster also consumed more of their daily energy from protein than the other clusters (17.8 vs 16.4% in the ‘Whole milk' cluster and 16.5% in the ‘Butter and cream' cluster). The total milk consumption was highest in the ‘Reduced fat milks and yogurt' cluster, at 33 g per MJ energy intake, but this figure was mostly due to skimmed and semi-skimmed milk, whereas whole-milk intake was very low in this cluster. Total cheese intake was similar across the clusters, but whole-milk drinkers consumed more of the higher-fat cheeses than the ‘Butter and cream' cluster or the ‘Reduced fat milks and yogurt' cluster (1.8 g per MJ vs 0.8 and 1.1 g), whereas the ‘Reduced fat milks and yogurt' cluster consumed more of the lower-fat cheeses.

To examine the rest of the diet consumed within these clusters, the non-dairy foods consumed were grouped into one of eight food groups, and the percentage contribution to energy from each food group was calculated in each of the three clusters (Supplementary Table S4). There was no difference across the clusters in the percentage energy derived from ‘Biscuits, cakes & pastries' or from ‘Savoury snacks and confectionary' or ‘Meat, fish and their dishes' (after adjustment for multiple comparisons). ‘Rice, grains, breads and cereals' were higher in the ‘Reduced fat milks and yogurt' cluster. Percent energy from ‘Beverages' and ‘Potato products' was higher in the whole-milk group. Percent energy from ‘Fruit and vegetables' was higher in the ‘Reduced fat milks and yogurt' cluster, as was the energy from ‘Milk, cheese and yogurt'. Percent energy from ‘Dairy, and dairy-containing recipes' was higher in the ‘Butter and cream cluster' (Supplementary Table S4). Tertiles of individual dairy components were cross-tabulated with the dairy intake clusters (Supplementary Table S5), and *X*^2^ analyses verified that the three clusters were significantly different in terms of their spread across the tertiles, demonstrating distinctly different patterns of dairy food intakes and supporting the use of clustering to identify patterns of dairy food intake.

A HEI variable, based on published dietary indices,^41^ was examined in relation to dairy tertiles, and to dairy food clusters, adjusting for age, gender and energy intake. The score did not differ across tertiles of total dairy, yet when the clusters were examined, there was a significant difference in the HEI score across clusters (*P*<0.001) (Table 3). The ‘Reduced fat milks and Yogurt' cluster had a significantly higher mean HEI score, and significantly lower SFA intakes than the other clusters (*P*<0.001) (Table 3). Despite this, there were few differences observed in the markers of metabolic health across the different dairy clusters (Table 3) —TNF-α was significantly higher in the ‘Whole milk' cluster compared with the other clusters (*P*=0.018)— serum triglycerides and total cholesterol were lower in the ‘Whole milk' and ‘Butter and cream' clusters than in the ‘Reduced milk and yogurt' cluster (*P*=0.028 and *P*=0.015, respectively).

## Discussion

The aim of this analysis was to examine consumption of dairy food intakes and metabolic health, looking both at intakes of individual dairy foods, and patterns of dairy foods in the adult population of Ireland. Total dairy intake was associated with lower measures of body fatness, including waist-to-hip ratio. A similar relationship was observed for total milk consumption, and for yogurt, although no differences in various metabolic health markers were observed across levels of cheese intake. These results are supported by a recent review, where authors concluded that dairy consumption has a modest, positive effect with respect to body weight and body fatness.^45^ In this cohort, total dairy and total milk consumption (which included whole- and reduced fat milks) was associated with lower circulating levels of some inflammatory biomarkers, with greater levels of adiponectin, and with increased insulin sensitivity, after adjustment for BMI, age, gender, energy intake, social class and smoking. Conversely, IL10, an anti-inflammatory marker, trended towards being higher in the low-milk consumers. These findings are largely in agreement with a number of previous studies that have observed an association between dairy intakes and circulating inflammatory markers^46, 47, 48^ and points to a potential role for milk in metabolic syndrome prevention or management.

Total dairy intake was also associated with a reduction in blood pressure. This finding is in agreement with the findings of Livingstone *et al.,*^49^ where a prospective analysis on the Caerphilly study demonstrated that augmentation index, an measure of arterial stiffness, was lower in those with the greatest dairy intake. When individual foods were examined, high-milk consumers in that study had a 10.4 mm Hg lower systolic blood pressure than non-consumers of milk, whereas butter intake in the same cohort was associated with a greater systolic blood pressure, demonstrating the importance of examining the overall patterns of dairy food consumption in addition to individual foods. The lack of observations between blood pressure and dairy products other than milk observed within our study could potentially be due to milk being the most widely consumed dairy product and at the greatest quantities within this cohort, and therefore similar analyses should be performed in much larger studies to examine whether other dairy products are also implicated. Increased total yogurt consumption was associated with significantly lower levels of pro-inflammatory cytokine TNFA, a result that is keeping with studies showing that yogurt consumption, potentially via lactic acid bacterial stimulation, may modulate cytokine production.^50, 51, 52^ ‘Total yogurt' here included drinking yogurts, some of which could be classified as probiotic drinking yogurts, which may have partly accounted for this result.

Cheese consumption was not associated with any measures of body fatness, or with many of the markers of metabolic health in this cohort, although C-peptide was higher in the higher consumers of cheese, and QUICKI, a measure of insulin sensitivity, trended towards being higher in the higher cheese consumers. C-peptide is associated with insulin sensitivity; these results together suggesting that cheese intake could be associated with improved insulin sensitivity in this cohort. Cheese consumers also had greater percentage energy from fat and from SFA than non-consumers, although blood lipid profiles did not differ across tertiles of cheese intake. The absence of association between cheese consumption and blood lipid profiles observed within this analysis is also in agreement with several recent intervention studies, which suggest that the SFA, consumed within the matrix of cheese, may not adversely impact blood lipid profiles.^11, 13, 32, 53^ Differences in the calcium contents of different dairy products,^33^ and differences in sphingolipid content,^31^ are two of the hypotheses that have been put forward to explain these phenomena in previous studies. Further research is required to fully understand the underlying mechanisms of the differences between butter, cream, cheese and milk in their cholesterol-raising abilities.

To our knowledge, this paper represents the first analysis where patterns of dairy food intakes have been examined in relation to metabolic health markers. Three main patterns of dairy food consumption were observed in this cohort—whole-milk consumers, reduced fat milk and yogurt consumers, and cream and butter consumers. The high level of agreement between the two cluster analyses conducted (the first using all reporters and the second using acceptable reporters only) indicates that the clusters of dairy consumption identified here are relatively stable across reporting types in this cohort. Mean daily fat intakes (total and saturated) were significantly lower in the ‘Reduced fat milks and yogurt' cluster. However, unexpectedly, triglycerides and total cholesterol were higher in this group than in the ‘Butter and cream' and the ‘Whole milk' cluster. One possibility for this result is that these individuals may have been following advice to consume reduced fat dairy, perhaps in response to cholesterol concerns or other reasons. However, as the results presented were adjusted for age, gender, HEI score and BMI differences, which would also be associated with these risks, this seems unlikely. Another potential explanation is that this could be partly due to the higher sphingolipid content of cream,^54^ since as mentioned above, recent evidence suggests that this may affect the impact of the SFA on the blood lipid profile.^31^ Alternatively, the ‘Reduced fat milks and yogurt' consumers may also have been higher consumers of other foods not fully captured by the 11 food groupings used here, and it is possible that some other dietary factor could have resulted in the higher serum triglycerides and total cholesterol observed, such as the percentage energy from carbohydrate. The clusters were based on the patterns of actual intake, which were categorised based on fat content, whereas the tertiles did not distinguish between fat content. Individuals in the ‘Reduced fat milks and yogurt' cluster had significantly greater % energy from the food group ‘Rice, grains, breads and cereals' (Supplementary Table S4), and a higher triglyceride level. This is consistent with evidence that suggests that increased carbohydrate in the diet is associated with increases triglyceride levels, for example,^55^ which could explain this otherwise seemingly anomalous result.

Dietary pattern analysis offers considerable advantage over examining tertiles of consumption alone, as it examines intakes in the context of overall food intake, and allows for the identification of patterns rather than single foods or nutrients in isolation. Examining intakes via both methods within a cohort offers a more holistic overview of the impact of dairy intake and health markers.

A HEI variable, based on published dietary indices,^41^ was examined in relation to dairy tertiles, and to clusters, adjusting for age, gender and energy intake. The score did not differ across tertiles of total dairy, yet when the clusters were examined, there was a significant difference in the HEI score across clusters (Table 3), demonstrating that the cluster analysis captures a more encompassing image of dietary intakes than tertile analysis alone. It should also be noted that although the figures were adjusted for differences in the AHEI score in order to account for other aspects of the diet, the AHEI is based on multiple factors. For example, the HEI used here assigned a value for trans fat intake among others. However, the food source of that fat is not considered, meaning that this could result in higher healthy eating scores for those with lower dairy fat, although trans fat intakes were generally low overall. The adjustment, here, for HEI score may not have fully accounted for the differences in energy from carbohydrate.

Despite the differences observed for metabolic health markers across tertiles of individual dairy components, few differences were observed across the clusters, even after adjustment for the HEI. Of note, total cholesterol and triglycerides were higher in the ‘Reduced fat milk and yogurts' cluster. This was surprising, considering the tertile results for yogurt. Overall this suggests that although some dairy foods were associated with favourable outcomes when considered in isolation, when the patterns of intakes were considered in their entirety, the resultant blood lipid profiles from reduced fat dairy appear less favourable when eaten as part of a low-fat, high-carbohydrate dietary pattern. Recently, the link between SFA and metabolic health has been revisited, and these results would appear to agree with some of the most recent findings, as the ‘Butter and cream' and ‘Whole milk' clusters, despite having greater fat intakes and SFA intakes, do not have adverse blood lipid profiles. One limitation of the present work is the fact that fasting blood samples were not obtained for every subject, which left a much smaller cohort of individuals in which to examine biochemistry. Due to the differences in the number of participants that fall within the different dairy food clusters, it is important that this work be repeated in larger cohorts to determine whether these observations translate to other population groups. Another potential limitation of the work includes the fact that the AHEI score also includes percentage energy from trans fat as one of the nine components without accounting for the food source of the fat. However, if anything, as dairy fat is a significant source of dietary trans fat being able to account for the food source could have lead to an even more positive outcome for the higher-fat dairy groups, as they would have received a lower HEI score based on this component.

## Conclusion

This study applies the concept of dietary pattern analysis to understanding dairy food intakes and allows for the exploration of patterns of dairy food intakes with differing fat contents. Here we show that clear and robust patterns of dairy food intake exist in the Irish population. The results of the tertile analysis suggest that dairy foods overall may offer potential for weight management, particularly milk and yogurt. Dairy foods, principally milk, may also have a role in the control of blood pressure, and potentially in the management of blood glucose. Cheese consumption was not associated with adverse lipid profiles, measures of body fatness or other markers of metabolic health in this cohort. Although greater overall dairy food consumption, driven mainly by milk and yogurt, was associated with more favourable body weight status, no single pattern of dairy food consumption stood out as having an overall healthier profile in this reportedly healthy population sample, when actual patterns of intake were examined. In fact, a ‘Reduced fat milks and yogurt' pattern was associated with higher triglycerides. As this cluster consumed a lower percentage energy from fat, and a higher percentage energy from grains, this suggests that the food intake pattern associated with low fat high carbohydrate may be less healthy than other patterns. More research is needed to better understand this result.

The results presented here demonstrate the importance of considering not only intakes of discrete foods, but also the patterns in which they are consumed in the diet, particularly in relation to dairy food intake patterns. Due to the current debate over dietary sources of SFA, the application of this concept to larger data sets, including ‘at-risk' cohorts, is warranted.

## Figures and Tables

**Table 1:** Metabolic markers of health across tertiles of total dairy consumption

*Variable*	*Low (1.25–180.6 g)*		*Medium (181.3–323.2 g)*		*High (324.2–1630.0 g)*		P-*value*
	n	*Mean ±s.e.*	n	*Mean ±s.e.*	n	*Mean ±s.e.*	
BMI (kg m^−^^2^)	465	27.8^c^ ±4.6	476	26.8^c,d^±5.4	470	26.7^d^±4.9	**<0.001**
Body fat (%)	439	31.1^c^±0.7	442	27.6^d^±0.7	437	26.8^d^±0.5	**<0.001**
Muscle mass (kg)	435	51.6±0.6	440	51.4±0.6	435	50.4±0.4	0.195
Waist circumference (cm)	406	93.7^c^±11.0	428	91.0^d^±1.0	429	87.8^e^±13.4	**<0.001**
Waist-to-hip ratio	408	0.89^c^±0.01	427	0.88^d^±0.01	429	0.86^e^±0.1	**<0.001**
BP—systolic (mmHg)	425	126.4±0.7	446	125.6±0.8	430	123.3±0.8	**0.02**
BP—diastolic (mmHg)	427	80.0^c^±0.5	446	78.8^c^±0.5	430	76.4^d^±0.6	**<0.001**
Serum trigs (mmol l^−1^)	195	1.3±0.05	234	1.3±0.05	302	1.3±0.05	0.968
Serum total cholesterol (mmol l^−1^)	195	4.96±1.0	235	4.86±1.0	302	4.99±0.9	0.247
Serum direct HDL (mmol l^−1^)	194	1.6±0.02	233	1.6±0.02	300	1.6±0.03	0.238
LDL-C (calculated) (mmol l^−1^)	192	2.85±0.9	231	2.7±0.9	298	2.8±0.8	0.253
Serum glucose (mmol l^−1^)	147	5.18^c^±0.6	185	5.24^d^±0.06	223	5.34^d^±0.07	0.216
Serum insulin (μ IU ml^−1^)	149	10.56^c^±0.64	183	7.71^d^±0.52	222	7.67^d^±0.51	**0.001**
HOMA^a^	147	2.5±0.2	185	2.1±0.2	231	1.9±0.2	0.062
QUICKI^b^	147	0.35^c^±0.01	185	0.36^d^±0.01	231	0.37^d^±0.01	**0.001**
Serum IL2 (pg ml^−1^)	103	2.01±0.2	115	1.6±0.19	156	1.5±0.2	0.188
Serum IL6 (pg ml^−1^)	134	2.0±0.3	174	1.9±0.3	203	2.1±0.3	0.732
Serum IL10 (pg ml^−1^)	136	1.1±0.2	172	0.93±0.2	217	1.0±0.2	0.619
Serum leptin (ng ml^−1^)	118	5.7^c^±0.5	150	4.2^d^±0.4	159	3.9^d^±0.6	**0.033**
Serum resistin (ng ml^−1^)	145	6.2±0.2	185	6.0±0.2	219	5.9±0.3	0.782
Serum C-peptide (ng ml^−1^)	145	2.33^c^±0.2	181	1.8^d^±0.2	216	1.8^d^±0.2	**0.04**
Serum TNFA (pg ml^−1^)	145	7.1±0.2	185	6.72±0.2	219	6.6±0.2	0.501
Adiponectin (μg ml^−1^)	149	6.09^c^±0.3	188	5.8^c^±0.2	232	6.8^d^±0.3	**0.013**
Leptin soluble receptor (ng ml^−1^)	149	27.1^c^±0.5	189	28.1^d^±0.5	232	29.0^d^±0.5	**0.03**

Abbreviations: ANCOVA, analysis of covariance; BMI, body mass index; BP, blood pressure; HDL, high-density lipoprotein; HOMA, Homeostasis Model Assessment; IL, interleukin; LDL-C, low-density lipoprotein-cholesterol; QUICKI, Quantitative Insulin Sensitivity Check Index; SFA, saturated fat; TNFA, tumour necrosis factor alpha.

a HOMA was calculated by (Glucose x Insulin) /22.5.

b QUICKI was derived using the inverse of the sum of the logarithms of the fasting insulin and fasting glucose: different superscript (c, d, e) letters indicate significant differences between the groups after *post hoc* correction. Mean values were analysed across clusters via ANCOVA, adjusting for age, gender, energy intake, Healthy Eating Index, BMI, social class, % energy from SFA and smoking habits, where applicable. Bonferroni correction was applied during ANCOVA.

*n* are presented individually for each variable, as not all variables were available for all the subjects. Significant values *P* < 0.05 are shown in bold.

**Table 2:** Cluster characteristics—dairy intakes per MJ in the different clusters (*n*=1497) and %energy from macronutrients

*Variable*	*‘Whole milk' Cluster* n *675*	*‘Reduced fat milks and yogurt' Cluster* n *56z4*	*‘Butter and cream' cluster* n *258*	P-*value*
	*Mean ±s.e.*	*Mean ±s.e.*	*Mean ±s.e.*	
Mean daily saturated fat per g	32.2^a^±14.0	25.7^b^±11. 0	32.2^a^±11.8	**<0.001**
Mean daily total fat per g	80.6^a^±31.4	67.5^b^±26.0	80.7^a^±26.8	**<0.001**
% energy MUFA	12.7^a^±2.7	11.7^b^±2.7	12.6^a^±2.6	**<0.001**
% energy PUFA	5.9±2.1	6.1±2.5	5.9±1.8	0.46
% energy SFA	13.8^a^±3.5	12.2^b^±3.5	14.0^a^±3.3	**<0.001**
% Energy fat	34.7^a^±6.3	32.0^b^±6.6	34.9^a^±6.2	**<0.001**
% Energy protein	16.4^a^±3.4	17.8^b^±3.7	16.5^a^±3.8	**<0.001**
Age/years	43.5±17.1	45.7±16.9	44.5±17.2	0.074
Energy/MJ	8.7^a^±2.9	7.9^b^±2.6	8.8^a^±2.6	**<0.001**
Male:female ratio	58:42	41:59	46:54	**<0.001**
Total milk per mJ	26.7^a^±21.5	33.4^b^±21.8	23.9^a^±16.4	**<0.001**
Whole milk per mJ	21.5^a^±22.2	5.2^b^±8.7	11.1^c^±12.9	**<0.001**
Semi-skimmed milk per mJ	3.1^a^±7.4	17.3^b^±21.7	7.7^c^±13.5	**<0.001**
Fortified milk per mJ	0.4^a^±2.4	6.2^b^±14.2	2.7^c^±9.8	**<0.001**
Skimmed milk per mJ	0.6^a^±2.9	4.1^b^±11.6	1.7^a^±5.6	**<0.001**
Total cheese per mJ	2.2^a^±2.3	2.0^a^±2.1	1.8^b^±1.7	**0.011**
Lower-fat cheeses per mJ	0.4^a^±0.7	1.3^b^±1.8	0.6^a^±1.0	**<0.001**
Higher-fat cheeses per mJ	1.8^a^±2.1	0.8^b^±1.1	1.1^b^±1.5	**<0.001**
Skimmed milk cheese per mJ	0.0^a^±0.1	0.1^b^±0.7	0.0^a^±0.2	**0.002**
Partially skimmed milk cheese per mJ	0.0^a^±0.1	0.0^b^±0.3	0.0^a^±0.2	0.05
Medium-fat cheese per mJ	0.4^a^±0.7	1.1^b^±1.7	0.6^a^±0.9	**<0.001**
Full fat cheese per mJ	1.7^a^±2.1	0.7^b^±1.1	1.1^c^±1.4	**<0.001**
High-fat cheese per mJ	0.1±0.4	0.0±0.2	0.1±0.3	0.47
Butter per mJ	0.0^a^±0.1	0.0^a^±0.1	0.4^b^±0.4	**<0.001**
Total cream per mJ	0.1^a^±0.2	0.1^a^±0.3	1.1^b^±1.3	**<0.001**
Total yogurt per mJ	1.6^a^±3.3	7.3^b^±8.7	3.6^c^±5.4	**<0.001**

Abbreviations: ANCOVA, analysis of covariance; BMI, body mass index; HEI, healthy eating index; MUFA, monounsaturated fatty acids; PUFA, polyunsaturated fatty acids; SFA, saturated fat. Mean values were analysed across clusters of dairy intakes via ANCOVA adjusting for age, gender, energy intake, HEI score and BMI. Different superscript (a, b, c) letters indicate significant differences between the groups after *post hoc* correction (significance level of 0.0018 was applied after correction for multiple tests). Significant values *P* < 0.05 are shown in bold.

**Table 3:** Markers of metabolic health across clusters of dairy consumption

*Variable*	*Cluster 1 ‘Whole milk'*		*Cluster 2 ‘Reduced fat milks and yogurt'*		*Cluster 3 ‘Butter and cream'*		P*-value*
	n	*Mean±s.e.*	n	*Mean±s.e.*	n	*Mean±s.e.*	
Healthy Eating Index	488	23.3^c^±8.5	371	28.0^d^±10.0	189	25.0^e^±9.4	**<0.001**
BMI (kg m^−^^2^)	601	26.9±4.6	512	27.3±5.4	239	227.1±4.9	0.474
Body fat (%)	589	29.3±9.1	497	29.1±8.9	231	29.2±8.9	0.593
Muscle mass (kg)	400	50.8±11.0	301	52.3±11.2	161	51.4±11.1	0.205
Waist circumference (cm)	378	89.7±12.3	301	89.2±12.3	166	89.2±14.0	0.443
Waist-to-hip ratio	378	0.87±0.1	301	0.87±0.1	166	0.87±0.1	0.802
BP—systolic (mmHg)	249	123.41±1.0	205	125.42±1.2	164	120.6±1.6	0.053
BP—diastolic (mmHg)	249	78.2±10.7	205	77.7±10.5	105	76.9±10.8	0.338
Serum trigs (mmol l^−1^)	251	1.31^c,d^±0.05	212	1.36^c^±0.06	106	1.13^d^±0.07	**0.028**
Serum total cholesterol (mmol l^−1^)	264	4.94^c^ ±0.07	216	5.16^d^±0.06	109	4.8^c^±0.1	**0.015**
Serum direct HDL (mmol l^−1^)	262	1.54±0.02	214	1.62±0.03	108	1.57±0.04	0.126
LDL-C (calculated) (mmol l^−1^)	259	2.80±0.06	213	2.91±0.07	108	2.72±0.09	0.217
Serum glucose (mmol l^−1^)	261	5.30±0.06	216	5.23±0.07	109	5.12±0.09	0.225
Serum insulin (μ IU ml^−1^)	234	9.00±0.46	205	8.74±0.54	106	9.51±0.70	0.689
HOMA^a^	259	2.28±0.15	216	2.18±0.17	107	2.37±0.22	0.791
QUICKI^b^	259	0.36±0.002	216	0.36±0.003	107	0.36±0.004	0.573
Serum IL2 (pg ml^−1^)	240	1.73±0.16	195	1.51±0.2	94	1.68±0.25	0.689
Serum IL6 (pg ml^−1^)	240	1.96±0.23	195	2.13±0.27	94	1.52±0.35	0.372
Serum IL10 (pg ml^−1^)	240	0.92±0.15	200	0.96±0.17	103	1.1±0.21	0.739
Serum leptin (ng ml^−1^)	252	5.14±0.46	210	4.29±0.53	106	4.9±0.71	0.473
Serum resistin (ng ml^−1^)	362	6.04±0.19	325	5.91±0.23	148	6.13±0.29	0.816
Serum C-peptide (ng ml^−1^)	174	2.0±0.13	134	1.86±0.16	74	2.27±0.21	0.289
Serum TNFA (pg ml^−1^)	252	7.23±0.16	210	6.55^d^±0.19	106	6.82^d,e^±0.24	**0.023**
Adiponectin (μg ml^−1^)	263	5.89±0.20	216	6.30±0.24	109	5.85±0.30	0.342
Leptin soluble receptor (ng ml^−1^)	264	27.51±6.0	216	28.7±7.1	109	28.1 ±6.6	0.056

Abbreviations: ANCOVA, analysis of covariance; BMI, body mass index; BP, blood pressure; HDL, high-density lipoprotein; HOMA, Homeostasis Model Assessment; IL, interleukin; LDL-C, low-density lipoprotein-cholesterol; QUICKI, Quantitative Insulin Sensitivity Check Index; SFA, saturated fat.

a HOMA was calculated by (Glucose x Insulin) /22.5.

b QUICKI was derived using the inverse of the sum of the logarithms of the fasting insulin and fasting glucose: mean values were analysed across clusters via ANCOVA, adjusting for age, gender, energy intake, Healthy Eating Index, smoking and BMI, where applicable (i.e., not line 1 or 2). Different superscript (c, d, e) letters indicate Bonferroni correction was applied during ANCOVA. Significant differences between the groups after *post hoc* correction.

*Ns* are presented individually for each variable, as not all variables were available for all the subjects. Figures are shown for fasted samples only. Significant values *P* < 0.05 are shown in bold.
