# Supplementary material for: Patterns of dairy food intake, body composition and markers of metabolic health in Ireland: results from the National Adult Nutrition Survey
Source: Nutr Diabetes. 2017 Feb 20;7(2):e243–. doi: 10.1038/nutd.2016.54 (PMC5360859; doi:10.1038/nutd.2016.54)
Supplement: Supplementary Tables [file nutd201654x1.docx]

**Supplementary Tables**

**Supplementary Table S1. Markers of metabolic health across tertiles of total milk consumption (adjusted)**

|  | Low | | | | Medium | | | High | | |
| --- | --- | --- | --- | --- | --- | --- | --- | --- | --- | --- |
| Variable | *n* | Mean ±SE | *n* | Mean ±SE | | *n* | Mean ±SE | | *P* |  |
| Milk (g/day) | 499 | 167.97 ±11.58 | 499 | 308 ±12.17 | | 499 | 397.49 ±13.67 | | **-** |  |
| BMI (kg/m^2^) | 455 | 27.56 ^a^ ±5.6 | 445 | 26.96 ^b^±4.61 | | 452 | 26.73b ±4.61 | | **0.008** |  |
| Body fat (%) | 444 | 30.45 ±9.07 | 434 | 29.64 ±8.57 | | 439 | 27.46 ±9.21 | | 0.076 |  |
| Muscle Mass (kg) | 442 | 50.94 ±11.13 | 433 | 51.16 ±10.93 | | 436 | 53.45 ±11.19 | | 0.065 |  |
| Waist circumference (cm) | 418 | 91.92 ±0.3 | 415 | 91.45 ±0.3 | | 431 | 92.05 ±0.3 | | 0.127 |  |
| Waist-to-hip ratio | 418 | 0.88 ±0.003 | 415 | 0.88 ±0.003 | | 430 | 0.87 ±0.003 | | 0.136 |  |
| BP – systolic (mmMg) | 432 | 125.63 ±0.7 | 433 | 124.7 ±0.69 | | 437 | 124.33 ±0.71 | | 0.423 |  |
| BP – diastolic (mmMg) | 432 | 79.16^a^ ±0.48 | 433 | 78.36^b^ ±0.47 | | 437 | 76.72^c^ ±0.48 | | **0.002** |  |
| Serum triglycerides (mmol/L) | 277 | 1.31 ±0.04 | 274 | 1.26 ±0.04 | | 297 | 1.21 ±0.04 | | 0.251 |  |
| Serum total cholesterol (mmol/L) | 278 | 4.94 ±0.06 | 274 | 4.96 ±0.06 | | 297 | 4.94 ±0.06 | | 0.789 |  |
| Serum direct HDL (mmol/L) | 273 | 1.55 ±0.02 | 273 | 1.55 ±0.02 | | 295 | 1.55 ±0.02 | | 0.998 |  |
| LDL-C (Calculated) (mmol/L) | 270 | 2.8 ±0.05 | 271 | 2.83 ±0.05 | | 295 | 2.83 ±0.05 | | 0876 |  |
| Serum glucose (mmol/L) | 276 | 5.26 ±0.06 | 273 | 5.23 ±0.06 | | 297 | 5.23 ±0.06 | | 0.929 |  |
| Serum Insulin (uIU/mL) | 277 | 10.13 ±0.41 | 272 | 8.71 ±0.41 | | 295 | 8.73 ±0.4 | | 0.02 |  |
| Homeostasis Model assessment (HOMA) | 275 | 2.5 ±0.14 | 270 | 2.11 ±0.14 | | 295 | 2.14 ±0.13 | | 0.08 |  |
| Quantitative Insulin Sensitivity Check Index (QUICKI) | 275 | 0.35^a^ ±0.002 | 270 | 0.36 ^a,b^ ±0.002 | | 295 | 0.36 ^b^ ±0.002 | | **0.002** |  |
| Serum IL-2 (pg/mL) | 176 | 1.9 ±0.13 | 178 | 1.63 ±0.13 | | 187 | 1.49 ±0.13 | | 0.087 |  |
| Serum IL-6 (pg/mL) | 248 | 2.31 ±0.31 | 249 | 1.88 ±0.3 | | 271 | 2.1 ±0.3 | | 0.610 |  |
| Serum IL-10 (pg/mL) | 249 | 1.13 ±0.11 | 256 | 0.81 ±0.11 | | 274 | 0.87 ±0.11 | | 0.076 |  |
| Serum Leptin (ng/mL) | 228 | 5.7 ±0.41 | 221 | 4.79 ±0.4 | | 214 | 5.52 ±0.41 | | 0.228 |  |
| Serum Resistin (ng/mL) | 267 | 6.08 ±0.17 | 266 | 6.16 ±0.17 | | 283 | 6 ±0.17 | | 0.742 |  |
| Serum C-Peptide (ng/mL) | 265 | 2.4 ±0.12 | 263 | 2 ±0.12 | | 279 | 1.97 ±0.12 | | **0.024** |  |
| Serum TNFA (pg/mL) | 267 | 6.83 ±0.14 | 266 | 6.82 ±0.14 | | 283 | 6.7 ±0.14 | | 0.768 |  |
| Adiponectin (ug/mL) | 278 | 6.0 ±0.17 | 274 | 6.07 ±0.17 | | 297 | 6.06 ±0.17 | | 0.933 |  |
| Leptin soluble receptor (ng/mL) | 278 | 27.1±0.38 | 275 | 27.45 ±0.37 | | 297 | 27.7 ±0.37 | | **0.481** |  |

Due to the very small sample size of non-consumers, this table shows consumers only of total milk

*n*s are presented individually for each variable, since not all variables were available for all subjects

Mean values were analysed across tertiles via ANCOVA adjusting for age, gender, energy intake and BMI (HEI score did not differ across the tertiles and adjusting did not alter the outcomes)

Different superscript ^( a,b,c)^ letters indicate significant differences between groups after post hoc correction

**Supplementary Table S2 Markers of metabolic health across tertiles of total cheese consumption (adjusted)**

| Cheese tertiles, adjusted values | **Non consumers** | | **Low** | | **Medium** | | **High** | |  |
| --- | --- | --- | --- | --- | --- | --- | --- | --- | --- |
|  | ***n*** | Mean ±SE | ***n*** | Mean ±SE | ***n*** | **Mean** | ***n*** | **Mean** | ***p*** |
| Cheese (g/day) | 353 | - | 385 | 7.09 ±1.73 | 380 | 18.58 ±2.04 | 382 | 45.06 ±4.53 | - |
| BMI (kg/m^2^) | 191 | 26.06 ±0.31 | 219 | 26.27 ±0.29 | 259 | 26.25 ±0.26 | 292 | 26.23 ±0.26 | 0.960 |
| % body fat | 291 | 30.89 ±8.95 | 344 | 30.16 ±8.66 | 341 | 28.81 ±8.9 | 341 | 27.13 ±9.23 | 0.462 |
| Muscle mass (kg) | 289 | 50.94 ±11.45 | 343 | 50.68 ±11.07 | 338 | 50.82 ±10.82 | 341 | 54.81 ±10.74 | 0.863 |
| Waist circumference (cm) | 287 | 91.06 ±0.37 | 334 | 91.84 ±0.33 | 323 | 91.38 ±0.34 | 320 | 91.54 ±0.35 | 0.444 |
| Waist-to-hip ratio | 287 | 0.89 ±0.08 | 333 | 0.88 ±0.09 | 323 | 0.87 ±0.09 | 320 | 0.88 ±0.08 | 0.545 |
| BP – systolic (mmMg) | 296 | 125.7 ±0.86 | 344 | 124.45 ±0.78 | 332 | 125.22 ±0.79 | 330 | 124.28 ±0.83 | 0.592 |
| BP – diastolic (mmMg) | 296 | 77.83 ±0.59 | 344 | 77.98 ±0.53 | 332 | 78.84 ±0.54 | 330 | 77.62 ±0.57 | 0.413 |
| Serum triglyceride (mmol/L) | 174 | 1.17 ±0.05 | 227 | 1.28 ±0.05 | 220 | 1.28 ±0.05 | 227 | 1.29 ±0.05 | 0.322 |
| Serum total cholesterol (mmol/L) | 174 | 4.88 ±0.08 | 228 | 4.92 ±0.7 | 220 | 4.97 ±0.66 | 227 | 5 ±0.07 | 0.661 |
| Serum direct HDL (mmol/L) | 174 | 1.54 ±0.03 | 225 | 1.55 ±0.03 | 216 | 1.55 ±0.03 | 226 | 1.57 ±0.03 | 0.910 |
| Calculated LDL (mmol/L) | 174 | 2.79 ±0.06 | 225 | 2.78 ±0.06 | 213 | 2.83 ±0.06 | 224 | 2.85 ±0.06 | 0.861 |
| Serum glucose (mmol/L) | 174 | 5.21 ±0.07 | 227 | 5.97 ±0.06 | 218 | 5.22 ±0.06 | 227 | 5.23 ±0.67 | 0.787 |
| Serum insulin (uIU/mL) | 174 | 8.19 ±0.52 | 226 | 9.22 ±0.45 | 219 | 9.29 ±0.46 | 225 | 9.88 ±0.48 | 0.135 |
| Homeostasis model assessment (HOMA) | 173 | 2.02 ±0.17 | 225 | 2.23 ±0.15 | 217 | 2.25 ±0.15 | 225 | 2.47 ±0.16 | 0.329 |
| Quantitative insulin sensitivity check index (QUICKI) | 173 | 0.36 ±0 | 225 | 0.35 ±0 | 217 | 0.36 ±0 | 225 | 0.36 ±0 | 0.051 |
| Serum IL2 (pg/mL) | 106 | 1.5 ±0.17 | 149 | 1.83 ±0.14 | 151 | 1.58 ±0.13 | 135 | 1.73 ±0.16 | 0.408 |
| Serum IL6 (pg/mL) | 159 | 2.38 ±0.34 | 207 | 1.92 ±0.34 | 203 | 2.46 ±0.34 | 199 | 1.7 ±0.36 | 0.362 |
| Serum IL10 (pg/mL) | 164 | 0.9 ±0.14 | 208 | 1.1 ±0.12 | 201 | 1.1 ±0.12 | 206 | 0.76 ±0.13 | 0.289 |
| Serum Leptin (ng/mL) | 136 | 5.11 ±0.52 | 188 | 5.24 ±0.46 | 175 | 4.93 ±0.46 | 164 | 6.28 ±0.47 | 0.173 |
| Serum Resistin (ng/mL) | 171 | 5.78 ±0.21 | 215 | 5.98 ±0.19 | 214 | 6.17 ±0.19 | 216 | 6.35 ±0.2 | 0.255 |
| Serum C-peptide (ng/mL) | 169 | 1.59 ^a^ ±0.15 | 214 | 2.39 ^b^ ±0.2 | 211 | 2.7 ^b^ ±0.2 | 213 | 2.8 ^b^ ±0.2 | <0.001 |
| Serum TNFA (pg/mL) | 171 | 6.47 ±0.18 | 215 | 67.04 ±0.16 | 214 | 6.86 ±0.16 | 216 | 6.78 ±0.17 | 0.123 |
| Adiponectin (ug/mL) | 175 | 6.12 ±0.22 | 228 | 5.86 ±0.19 | 219 | 6.03 ±0.19 | 227 | 6.23 ±0.2 | 0.573 |
| Leptin soluble receptor (ng/mL) | 175 | 27.38 ±0.48 | 228 | 27.18 ±0.42 | 220 | 27.05 ±0.42 | 227 | 28.18 ±0.44 | 0.250 |
|  |  |  |  |  |  |  |  |  |  |

*n*s are presented individually for each variable, since not all variables were available for all subjects

Mean values were analysed across tertiles via ANCOVA adjusting for age, gender, energy intake, and BMI, where applicable. Smoking habits, social class and HEI score did not vary across groups, and adjusting with these did not affect the outcomes.

Different superscript ^( a,b,c)^ letters indicate significant differences between groups after post hoc correction

Bonferroni correction was not performed at a family level, due to the association of dairy consumption with a range of metabolic markers being an a-priori hypothesis

**Supplementary Table S3. Markers of metabolic health across tertiles of total yogurt consumption (adjusted)**

| Yogurt tertiles, adjusted values | **Non consumers** | | | | **Low** | | | **Medium** | | | | **High** |  |
| --- | --- | --- | --- | --- | --- | --- | --- | --- | --- | --- | --- | --- | --- |
|  | ***n*** | Mean ±SE | ***n*** | Mean ±SE | | ***n*** | Mean ±SE | | ***n*** | Mean ±SE | ***P*** | |  |
| Yogurt (g/day) | 809 | - | 229 | 21.7 ±0.74 | | 231 | 56.4 ±0.9 | | 228 | 131.2 ±3.73 | - | |  |
| BMI (kg/m^2^) | 713 | 27.36 ±0.19 | 210 | 26.87 ±0.34 | | 222 | 26.4 ±0.33 | | 207 | 27.04 ±34 | 0.391 | |  |
| % body fat | 692 | 29.12 ±0.14 | 207 | 29.62^a^ ±0.26 | | 220 | 29.67^a^ ±0.25 | | 198 | 28.41^b^ ±0.26 | **0.001** | |  |
| Muscle mass (kg) | 689 | 51.57 ±0.18 | 207 | 52.42 ±0.32 | | 218 | 51.81 ±0.32 | | 197 | 52.26 ±0.33 | 0.071 | |  |
| Waist circumference (cm) | 653 | 91.8^a^ ±0.23 | 200 | 91.74^a,b^ ±0.43 | | 210 | 91.23^a,b^ ±0.42 | | 201 | 90.36 ^b^ ±0.42 | **0.025** | |  |
| Waist-to-hip ratio | 652 | 0.88 ^a^ ±0.002 | 200 | 0.88 ^a,b^ ±0.004 | | 210 | 0.87^a,b^ ±0.004 | | 201 | 0.87 ^b^ ±0.004 | **0.013** | |  |
| BP – systolic (mmMg) | 685 | 125.05 ±0.53 | 205 | 123.98 ±1.01 | | 218 | 125.47 ±0.98 | | 194 | 124.63 ±1.04 | 0.724 | |  |
| BP – diastolic (mmMg) | 685 | 78.46 ±0.38 | 205 | 77.78 ±0.69 | | 218 | 78.11 ±0.67 | | 194 | 76.99 ±0.71 | 0.332 | |  |
| Serum triglyceride (mmol/L) | 439 | 1.35 ±0.03 | 138 | 1.27 ±0.06 | | 147 | 1.3 ±0.05 | | 124 | 1.19 ±0.06 | 0.073 | |  |
| Serum total cholesterol (mmol/L) | 439 | 4.89 ±0.05 | 138 | 5.01 ±0.09 | | 148 | 5 ±0.08 | | 124 | 4.95 ±0.09 | 0.480 | |  |
| Serum direct HDL (mmol/L) | 436 | 1.55 ±0.02 | 137 | 1.54 ±0.03 | | 144 | 1.52 ±0.03 | | 124 | 1.59 ±0.03 | 0.526 | |  |
| Calculated LDL (mmol/L) | 432 | 2.75 ±0.04 | 137 | 2.92 ±0.07 | | 143 | 2.9 ±0.06 | | 124 | 2.85 ±0.07 | 0.123 | |  |
| Serum glucose (mmol/L) | 437 | 5.22 ±0.05 | 138 | 5.38 ±0.08 | | 147 | 5.26 ±0.08 | | 124 | 5.23 ±0.09 | 0.454 | |  |
| Serum insulin (uIU/mL) | 433 | 9.58 ±0.32 | 138 | 8.83 ±0.58 | | 148 | 9.05 ±0.55 | | 125 | 8.9 ±0.6 | 0.577 | |  |
| Homeostasis model assessment (HOMA) | 431 | 2.33 ±0.11 | 138 | 2.19 ±0.19 | | 147 | 2.24 ±0.18 | | 124 | 2.27 ±0.2 | 0.922 | |  |
| Quantitative insulin sensitivity check index (QUICKI) | 431 | 0.35 ±0 | 138 | 0.36 ±0 | | 147 | 0.36 ±0 | | 124 | 0.36 ±0 | 0.176 | |  |
| Serum IL2 (pg/mL) | 277 | 1.67 ±0.1 | 90 | 1.56 ±0.18 | | 93 | 1.64 ±0.18 | | 81 | 1.68 ±0.19 | 0.948 | |  |
| Serum IL6 (pg/mL) | 407 | 2.33 ±0.24 | 122 | 1.7 ±0.44 | | 128 | 2.33 ±0.42 | | 111 | 1.58 ±0.46 | 0.341 | |  |
| Serum IL10 (pg/mL) | 397 | 0.98 ±0.09 | 130 | 0.77 ±0.15 | | 136 | 1.14 ±0.15 | | 116 | 0.84 ±0.16 | 0.302 | |  |
| Serum Leptin (ng/mL) | 333 | 5.28 ±0.32 | 118 | 6.02 ±0.57 | | 110 | 5.1 ±0.58 | | 102 | 5 ±0.6 | 0.562 | |  |
| Serum Resistin (ng/mL) | 424 | 6.13 ±0.13 | 135 | 6.02 ±0.24 | | 140 | 5.99 ±0.23 | | 117 | 6.06 ±0.22 | 0.90 | |  |
| Serum C-peptide (ng/mL) | 418 | 2.2 ±0.1 | 134 | 1.95 ±0.17 | | 140 | 2.32 ±0.16 | | 115 | 1.96 ±0.18 | 0.263 | |  |
| Serum TNFA (pg/mL) | 424 | 7.09^a^ ±0.11 | 135 | 6.66^a,b^ ±0.2 | | 140 | 6.43 ^b^ ±0.19 | | 117 | 6.20 ^b^ ±0.21 | **<0.001** | |  |
| Adiponectin (ug/mL) | 438 | 5.98 ±0.13 | 138 | 5.8 ±0.24 | | 148 | 5.91 ±0.23 | | 125 | 6.42 ±0.25 | 0.294 | |  |
| Leptin soluble receptor (ng/mL) | 439 | 27.21 ±0.3 | 138 | 27.64 ±0.54 | | 148 | 27.09 ±0.51 | | 125 | 28.4 ±0.55 | 0.264 | |  |

*n*s are presented individually for each variable, since not all variables were available for all subjects

Mean values were analysed across tertiles via ANCOVA adjusting for age, gender, energy intake, HEI score, and BMI, where applicable. Smoking habits and social class did not vary across groups, and adjusting with these did not affect the outcomes.

Different superscript ^( a,b,c)^ letters indicate significant differences between groups after post hoc correction

Bonferroni correction was not performed at a family level, due to the association of dairy consumption with a range of metabolic markers being an a-priori hypothesis

**Supplementary Table S4. Percentage Contribution to Energy From Food Groups across the Dairy Clusters**

|  | Percentage Contribution to Energy | | | |
| --- | --- | --- | --- | --- |
|  | Cluster 1  ‘Whole milk ’ | Cluster 2  ‘Reduced fat milk and yogurt’ | Cluster 3  ‘Cream and butter’ |  |
| Food Group | Mean ±SD | Mean ±SD | Mean ±SD | ***P*** |
| Rice, grains, breads & cereals | 19.6^a^ ±8.4 | 21.1^b^ ±8.7 | 18.4^a^ ±7.8 | **<0.001** |
| Biscuits, cakes & pastries | 5.2 ±5.7 | 5.2 ±5.2 | 5.7 ±6 | 0.331 |
| Savoury snacks & confectionary | 6.2 ±5.5 | 5.7 ±5.4 | 6.1 ±6 | 0.233 |
| Beverages | 8.2^a^ ±9.4 | 6.5^b^ ±7.8 | 7.2^a^ ±8.1 | 0.002 |
| Potato & potato products | 8.0^a^ ±5.7 | 6.6^b^ ±4.9 | 6.9^b^ ±4.7 | **<0.001** |
| Fruit & vegetables | 6.2^a^ ±4.7 | 8.0 ^b^ ±5.4 | 7.6^b^ ±4.9 | **<0.001** |
| Meat fish & their dishes | 19.4 ^a^ ±8.9 | 18.6 ^a,b^ ±8.3 | 18.0^b^ ±8.7 | 0.045 |
| Other foods (eggs, egg dishes, fats, oils, nuts, seeds) | 9.0 ^a^ ±5.8 | 8.1^a^ ±5.3 | 9.6^b^ ±5.8 | **0.001** |
| Dairy, and dairy-containing recipes | 18.1^a^ ±10.7 | 20.0 ^b^ ±10.7 | 20.4^b^ ±10.3 | **0.003** |

**Supplementary Table S5. Cross-tabulation of individuals within dairy food tertiles and individuals within the Dairy Clusters**

|  |  | Clusters | | |  |
| --- | --- | --- | --- | --- | --- |
| Tertiles (based on g per MJ) |  | Whole Milk, *n* | Reduced Fat Milks and Yoghurt, *n* | Cream and Butter, *n* | *X^2^* |
| Dairy, *n* 1497 | Low | 278 | 122 | 96 | *X^2^* = 57.04,  ***p*<0.001** |
|  | Medium | 207 | 213 | 80 |  |
|  | High | 189 | 229 | 83 |  |
| Full fat milks, *n* 1188 | Low | 105 | 212 | 79 | *X^2^* = 208.6, ***p*<0.001** |
|  | Medium | 195 | 115 | 86 |  |
|  | High | 293 | 45 | 58 |  |
| Reduced Fat milks, *n* 894 | Low | 164 | 75 | 59 | *X^2^* = 222.3, ***p*<0.001** |
|  | Medium | 69 | 163 | 66 |  |
|  | High | 21 | 242 | 35 |  |
| Higher fat cheeses, *n* 880 | Low | 103 | 127 | 63 | *X^2^* = 78.4; ***p<*0.001** |
|  | Medium | 135 | 102 | 57 |  |
|  | High | 206 | 49 | 38 |  |
| Lower fat cheeses, *n* 706 | Low | 117 | 74 | 44 | *X^2^* = 73.8; ***p*<0.001** |
|  | Medium | 108 | 86 | 42 |  |
|  | High | 44 | 156 | 35 |  |
| Yogurt, *n* 688 | Low | 91 | 84 | 54 | *X^2^* = 75.7; ***p*<0.001** |
|  | Medium | 78 | 111 | 41 |  |
|  | High | 25 | 171 | 33 |  |
| Cream, *n 313* | Low | 53 | 34 | 17 | *X^2^* = 160.1; ***p*<0.001** |
|  | Medium | 33 | 45 | 27 |  |
|  | High | 3 | 3 | 98 |  |
| Butter, *n 310* | Low | 54 | 32 | 17 | *X^2^* = 142.0; ***p*<0.001** |
|  | Medium | 24 | 16 | 64 |  |
|  | High | 2 | 0 | 101 |  |

*n*, number of consumers for each individual dairy food category, over the 4-d period. The tertiles shown here are based on g per MJ energy intake, as per creation of the clusters, to more accurately reflect the spread of dairy food intakes across clusters.
